# Mirnacle: machine learning with SMOTE and random forest for improving selectivity in pre-miRNA ab initio prediction

**DOI:** 10.1186/s12859-016-1343-8

**Published:** 2016-12-15

**Authors:** Yuri Bento Marques, Alcione de Paiva Oliveira, Ana Tereza Ribeiro Vasconcelos, Fabio Ribeiro Cerqueira

**Affiliations:** 1 Department of Informatics, Universidade Federal de Viçosa, Viçosa, 36570-900 Brazil; 2 Instituto Federal do Norte de Minas, Rua Mocambi, Teófilo Otoni, 39800-430 Brazil; 3 Department of Computer Science, University of Sheffield, Western Bank S10 2TNSheffield, UK; 4 Laboratório Nacional de Computação Científica, Rua Getúlio Vargas 333, Petropólis, 25651-071 Brazil

**Keywords:** Pre-miRNA ab initio prediction, Random forest, Smote, microRNA, Machine learning, Data mining

## Abstract

**Background:**

MicroRNAs (miRNAs) are key gene expression regulators in plants and animals. Therefore, miRNAs are involved in several biological processes, making the study of these molecules one of the most relevant topics of molecular biology nowadays. However, characterizing miRNAs in vivo is still a complex task. As a consequence, in silico methods have been developed to predict miRNA loci. A common ab initio strategy to find miRNAs in genomic data is to search for sequences that can fold into the typical hairpin structure of miRNA precursors (pre-miRNAs). The current ab initio approaches, however, have selectivity issues, i.e., a high number of false positives is reported, which can lead to laborious and costly attempts to provide biological validation. This study presents an extension of the ab initio method miRNAFold, with the aim of improving selectivity through machine learning techniques, namely, random forest combined with the SMOTE procedure that copes with imbalance datasets.

**Results:**

By comparing our method, termed Mirnacle, with other important approaches in the literature, we demonstrate that Mirnacle substantially improves selectivity without compromising sensitivity. For the three datasets used in our experiments, our method achieved at least 97% of sensitivity and could deliver a two-fold, 20-fold, and 6-fold increase in selectivity, respectively, compared with the best results of current computational tools.

**Conclusions:**

The extension of miRNAFold by the introduction of machine learning techniques, significantly increases selectivity in pre-miRNA ab initio prediction, which optimally contributes to advanced studies on miRNAs, as the need of biological validations is diminished. Hopefully, new research, such as studies of severe diseases caused by miRNA malfunction, will benefit from the proposed computational tool.

## Background

MicroRNAs (miRNAs) constitute currently a major research topic in molecular biology [1]. These molecules are responsible for post-transcriptional regulation of gene expression, and are involved in several biological processes such as embryonic differentiation [2], skeletal muscle development [3], several cardiovascular disorders [4], expansion of skin stem cells [5], hematopoiesis [6], control of proliferation and death of cells [7], insulin secretion [8], adipogenesis and obesity [9], diseases such as cancer [10], in addition to play relevant physiological roles in animals and plants [11].

In miRNA biogenesis, the corresponding gene is transcribed into a primary miRNA (pri-miRNA) that undergoes cleavage by the Drosha complex, resulting in a hairpin-shaped miRNA precursor (pre-miRNA) of approximately 70 nt. Next, the precursor is exported to the cytoplasm by the protein Exportin5 and cleaved into the mature miRNA by the enzyme Dicer [1].

Identifying miRNA loci in vivo is an expensive and complex task. As a result, computational tools have been developed to perform in silico predictions. Comparative approaches, i.e., methods that use conservation of functional genomic elements of phylogenetically-close species, are a natural means for carrying out predictions, because many miRNAs are well conserved among eukaryotes [12]. Additionally, methods based on next-generation sequencing (NGS) data are of increasing interest, as they deliver good sensitivity and provide the possibility of analyzing expression profiles [13]. Another important computational solution that complements the approaches mentioned above, and which is a good alternative for identifying species-specific miRNAs, is the so-called ab initio approach.

According to Tempel and Tahi [14], ab initio methods can be classified into three categories: 1) Methods whose input is a putative pre-miRNA sequence and that classify the given candidate as true or false; 2) methods whose input is comprised of a genomic sequence as well as some other information that helps to predict pre-miRNAs in the given sequence; 3) methods whose input is a genomic sequence and that use no external information to predict pre-miRNAs in the given sequence. Tempel and Tahi call the third category completely ab inito methods.

Ab initio methods are very useful when no closely-related species with identified miRNAs are available. Even when such an information is accessible, ab initio approaches are important for predicting pre-miRNAs that are specific to the studied genome. Furthermore, NGS still involves a considerable cost and laboratory infrastructure that are not the reality of a significant part of research groups worldwide. Our work matches the third category of ab initio methods. Some important approaches in this category have been previously proposed.

VMir was conceived to predict pre-miRNAs in viruses [15]. Initially, this method processes the input sequence through a sliding window to produce subsequences with length close to the expected length of a pre-miRNA. Then, in order to build a putative secondary structure for each subsequence, VMir uses the software RNAfold [16]. Finally, VMir calculates a score for each pre-miRNA candidate based on selected secondary structure attributes. Only those pre-miRNA candidates with scores above an informed threshold value are given as output.

CID-miRNA applies a stochastic context-free grammar built from secondary structures of known human pre-miRNAs, along with a J48 decision tree to predict pre-miRNAs in a given DNA sequence [17]. To learn the characteristics of negative cases in its model, CID-miRNA uses human ribosomal RNA.

Virgo predicts pre-miRNAs using a variant of the learning algorithm support vector machines (SVM) called SVM ^light^ [18, 19]. For training the classifier, validated human miRNA precursors are used as positive instances, while negative instances are artificially produced using coding regions of genes. Virgo extracts several subsequences from the input sequence, and computationally folds them with the software RNAfold. A filter is then applied to select the secondary structures that match known characteristics of pre-miRNAs. Finally, several attributes are extracted from the selected candidates so that the SVM model can assign them a final classification.

The method miRPara also applies SVM for performing classification and provides the prediction of mature miRNAs in addition to their precursors [20]. Initially, miRPara breaks the input sequence into 500-nt subsequences with a 200-nt overlap. Next, the subsequences are given as input to the software UNAFold for extracting hairpins [21]. Many of the obtained hairpins are then discarded by the application of a filter, and the remaining ones are further analyzed with UNAFold. At last, several attributes related to physical characteristics of miRNAs/pre-miRNAs are extracted from the final set of hairpins for predicting the most likely candidates. To construct the training sets, positive examples were extracted from validated miRNAs/pre-miRNAs, and negative examples were artificially produced from validated pri-miRNAs with the start position of their miRNAs randomly shifted within the sequences.

The method miRNAFold is based on a statistical approach defined from a previous analysis the authors made of pre-miRNAs contained in the miRBase database [14, 22]. Similarly to other methods, miRNAFold analyzes fixed-length sequence windows, searching for putative pre-miRNAs. For each window, miRNAFold constructs the most likely secondary structure without the use of third-party computational tools, in order to speed up the prediction procedure. This is accomplished by the construction of a base-pairing matrix for each window, which is analyzed in three stages. For each stage, several characteristics of parts of putative hairpins are observed according to the statistical study made previously. The final hairpins that match a certain percentage of these characteristics are given as likely pre-miRNA candidates.

The method of Titov and Vorozheykin (TVM) proposes a context-structural Markov model and a hidden Markov model for ab initio prediction of pre-miRNAs and miRNAs, respectively [23]. The authors used 1,872 human pre-miRNAs from the miRBase database as positive instances, and 1,872 random sequences from the pseudo-precursors constructed by Xue and colleagues as negative instances [24]. TVM uses the software GArna to produce secondary structures from the subsequences extracted from the input sequence [25]. Then, several patterns of the primary and secondary structures are evaluated with the proposed models.

Even though the methods described above have provided important contributions, they deliver very low selectivity (also known as precision), i.e., a high number of false positives (FPs) are produced, which means more work at the laboratory bench. In particular, the statistical study made for the method miRNAFold identified secondary structure characteristics of positive instances only, i.e., the method was based on pre-miRNAs alone. The authors did not analyze negative instances in their work. As a consequence, even reaching excellent sensitivity, large amounts of FPs are produced.

In this work, we propose an extension of miRNAFold, which includes negative examples and applies machine learning (ML) techniques to improve selectivity. We compared our approach with miRNAFold and all other methods mentioned above. Experiments with three datasets demonstrate that we preserved very high sensitivity, while substantially increasing selectivity. Our method, termed Mirnacle, provided an improvement of two-fold, 20-fold, and 6-fold in selectivity, for the respective datasets, compared with the best results of the other approaches.

## Methods

Our aim is to predict new pre-miRNAs from a DNA sequence without using any additional information (completely ab initio approach). For each subsequence extracted from the input sequence, Mirnacle executes three stages to try to build the most likely hairpin structure. Each stage classifies parts of the hairpin that is incrementally built. The steps are similar to the miRNAFold approach. One major difference is that we consider negative examples in addition to positive instances (previously identified pre-miRNAs). Another important distinction is the use of ML techniques to perform a more sophisticated classification, where each stage has its own model, with the aim of minimizing the number of false positives.

### Ab initio prediction

**Fig. 1:**
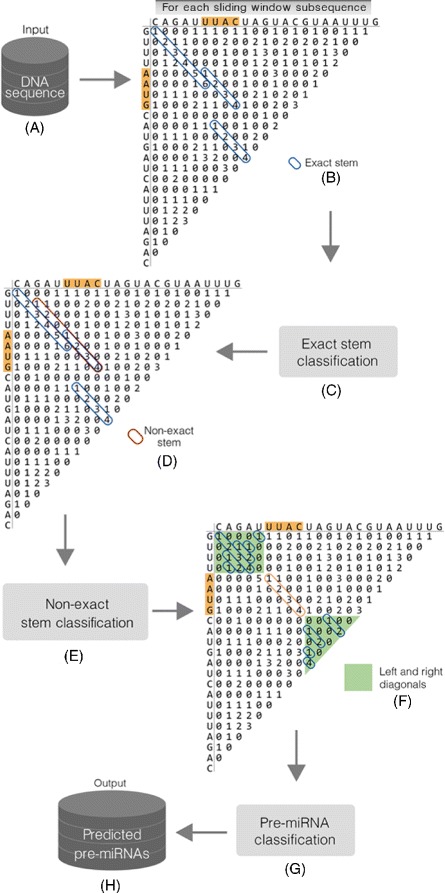
General view of the Mirnacle approach (Adapted from Tempel and Tahi [14]). Given the input DNA sequence **a**, a sliding window is used to extract subsequences of length close to the expected pre-miRNA length. For each subsequence, a triangular base pairing matrix (example for the sequence CAGAUUUACUAGUACGUAAUUUG) is constructed and analyzed in three stages. In the first stage (**b** and **c**), long exact stems (series of positive numbers in the diagonals) are sought and classified. Next, in the second stage (**d** and **e**), for each positively classified exact stem, its diagonal is searched to form a non-exact stem (series of positive numbers interspersed with series of 0’s) that also passes through a classification procedure. Finally, in the last stage (**f** and **g**), a complete hairpin is produced from each previously filtered non-exact stem, using the originally identified exact stem as the starting point for a further search in the matrix. In this search, other diagonals are tried so that secondary structures with asymmetrical internal loops are also considered. The resultant hairpins are then classified with a third ML model and only the ones predicted as positives are given as the final output (**h**)

Similarly to many methods in the ab initio category, Mirnacle applies a sliding window to the informed DNA sequence to obtain subsequences that may represent pre-miRNAs. For each extracted subsequence, the goal is to fold it into a typical pre-miRNA hairpin structure. Third-party programs are often used for this end. However, analogously to miRNAFold, putative foldings are represented here by a triangular base pairing matrix, and the most likely hairpin is obtained from a three-stages analysis of this matrix. Figure 1 illustrates the general structure of our method by showing an example of such a matrix.

To build the matrix for a given sequence *s*[0..*n*−1], a column *i* represents the character *s*[*i*], a row *i* represents the character *s*[*n*−1−*i*], and an entry *i,j* is a positive integer number if the corresponding bases are complementary, or zero, otherwise. The positive numbers indicate the extension of the paired region. Algorithm 1 clearly describes how the base pairing matrix is constructed. Lines 4-15 initialize the first column and the first row, while lines 16-24 fill the other entries.



**Fig. 2:**
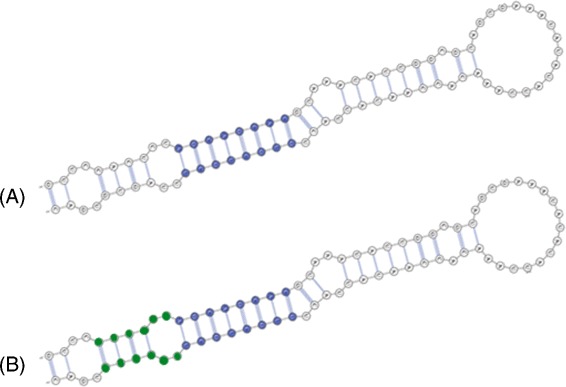
Illustration of the incremental approach performed in the base pairing matrix analysis. **a** A long exact stem (in blue) is identified (steps shown in Fig. 1 b/c). **b** The exact stem is then extended to a non-exact stem (Fig. 1 d/e, here in green and blue) that, in turn, is the basis to build a complete hairpin (procedure represented in Fig. 1 f/g)

The general idea is to build secondary structures incrementally. Minor parts of possible hairpins are identified and then extended to form the complete structures, as can be seen in Fig. 2. Initially, long regions of paired bases, termed exact stems, are sought (initial steps shown in Fig. 1 b/c and illustrated in Fig. 2 a). Next, the long exact stems found in the previous step are extended to non-exact stems, i.e., parts of a hairpin composed of paired bases interposed between unpaired regions (steps shown in parts d and e of Fig. 1 and depicted in Fig. 2 b). These unpaired regions are symmetrical loops whose size is less than the length of the surrounding exact stems. The extension of an exact stem is achieved by taking into account only its diagonal in the base pairing matrix. Each resultant non-exact stem is considered a good approximation of a hairpin and is thus used at the last stage as the basis for achieving a pre-miRNA secondary structure (the final steps shown in Fig. 1 f/g). For each non-exact stem, the exact stem that gave rise to it is fixed and other diagonals are explored to make possible the occurrence of asymmetrical internal loops (see Fig. 1 f).

Our main contribution here is the application of ML techniques with the objective of minimizing false positives. It contrasts with the verification of a list of criteria performed in miRNAFold. It is important to notice that each stage has its own ML model. For example, there is a specific model to apply on all exact stems of a minimum predefined length found in the first stage (Fig. 1 c). Only those instances considered as positives, i.e., for which the model assigns a probability value greater or equal to a predefined threshold, are given as input to the second stage. The non-exact stems produced from the positively classified exact stems, in turn, are classified with another ML model (Fig. 1 e), and only the instances regarded as positives pass to the next phase. In the last stage, a third ML model is used to classify (Fig. 1 g) the resultant hairpins to report the final predictions.

The three-stages procedure described above is repeated for each sliding window subsequence. At the end of each analysis, the sliding window is moved 10 nt downstream, as proposed by Tempel and Tahi [14]. This process continues until the end of the given DNA sequence.

### Datasets

To validate the proposed method, three datasets used in the experiments of miRNAFold were also used in our work to facilitate the comparisons. One of the datasets is an artificially constructed sequence obtained from 100 human pre-miRNAs interposed between human mRNAs, leading to a sequence of 30,500 nt. The other two datasets are real genomic data containing clusters of pre-miRNAs. The first one is a sequence extracted from the positive strand of the human chromosome 19, starting at position 54,169,933 and ending at position 54,485,651, containing 50 pre-miRNAs. The second sequence was obtained from the positive strand of the mouse chromosome 2, starting at position 10,388,290 and ending at position 10,439,906, comprehending 71 pre-miRNAs. These three datasets are referred throughout the text as HAD (human artificial data), HSD (*Homo sapiens* data), and MMD (*Mus musculus* data), respectively.

The sequences from zebrafish and sea squirt chromosomes used in the miRNAFold work were not included in our experiments because we could not match the stretches cited by the authors to the data available in GenBank. Furthermore, to our knowledge, there were no validated pre-miRNAs in the miRBase database for these two species at the moment we performed our experiments. Details about the datasets can be found in the work of Tempel and Tahi [14].

To build the learning models, three independent datasets to produce training sets (TSs) were constructed: TSHA, TSHS, and TSMM, one for each experiment with the three test sets: HAD, HSD, and MMD, respectively. Positive examples were obtained from experimentally validated pre-miRNA sequences present in miRBase release 21 [22]. For a fair model evaluation, all pre-miRNAs contained in the test sets were eliminated from TSHA, TSHS, and TSMM. Therefore, the positive instances in TSHA were a result of all validated human pre-miRNAs in miRBase subtracted by the pre-miRNAs present in HAD. Similarly, TSHS’s positive instances were obtained after subtracting the pre-miRNAs in HSD from the set of validated human pre-miRNAs. Finally, the positive instances of TSMM were the result of the validated mouse pre-miRNAs in miRBase minus the pre-miRNAs in MMD. As a consequence, TSHA, TSHS, and TSMM contain 235, 295, and 364 positive examples, respectively.

Negative examples, in turn, were comprised of other types of non-coding RNAs along with pseudo hairpins. Gene sequences of snRNA (small nuclear RNA), snoRNA (small nucleolar RNA), tRNA, and miscRNA (miscellaneous RNA) were extracted from GenBank and the NRDR repository [26], totaling 2,480 sequences from the *H. sapiens* genome and 3,298 sequences from the *M. musculus* genome. Moreover, 1,872 pseudo pre-miRNAs from the work of Xue et al. were considered to compose the *H. sapiens* TSs [24]. Therefore, TSHA, TSHS, and TSMM contain 4,352, 4,352, and 3,298 negative examples, respectively.

**Fig. 3:**
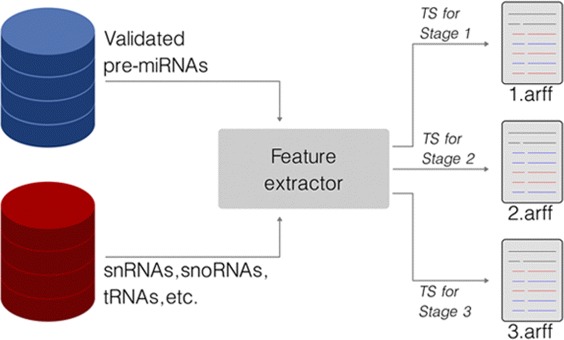
Training set construction. Each stage has its own training set for building its specific machine learning model. Real pre-miRNAs are used as positive examples, while negative examples are comprised of snRNAs, snoRNAs, tRNAs, miscRNAs, and pseudo hairpins

Notice that each stage has its own TS to build its particular model. Therefore, each of the datasets TSHA, TSHS, and TSMM led to three TSs that are identified along the text with the indexes 1, 2, and 3, e.g., the dataset TSHA gave rise to the TSs: TSHA1, TSHA2, and TSHA3, one for each respective stage. Figure 3 illustrates this procedure. In this work, the TSs are in the ARFF format. This is the native format of the Weka machine learning toolkit [27], whose application programming interface (API) is used in the Mirnacle implementation.

### Features

Another distinctive aspect of Mirnacle regarding miRNAFold was the inclusion of other features to improve the discriminatory power of the ML models. Besides the features proposed by Tempel and Tahi, we also considered dinucleotides, MFE (minimum free energy) 1, and MFE 2 from the work of Batuwita and Palade [28], as well as triplets from the work of Xue et al. [24]. Furthermore, the feature “size of the internal symmetric loop” was replace to other three features: Number of internal symmetric loops, average size of internal symmetric loops, and max size of internal symmetric loops. Still, the feature “size of the biggest side of the biggest bulge” was replaced to other two features: Size of the biggest bulge to the left of the terminal loop and size of the biggest bulge to the right of the terminal loop. Considering that dinucleotides and triplets mean 16 and 32 additional features, respectively, our approach includes 51 extra features. Notice that a triplet, according to Xue and colleagues [24], denotes whether each nucleotide in a consecutive sequence of three nucleotides is paired, represented by the symbol ‘(’, or unpaired, represented by the symbol ‘. ’, and also specifies the middle nucleotide. Therefore, the feature “U((.”, for example, means that there is a consecutive sequence of three nucleotides where the middle is U, the first and the second nucleotides are paired, and the third one is unpaired. The complete list of the features used in the ML model of each stage is given below:

- Features for exact stem classification in the first stage: Size, deltaG, percentage of each type of nucleotide, maximum number of consecutive nucleotides of each type, percentage of GU pairing, percentage of each possible dinucleotide, percentage of the difference between G+A and C+U, and percentage of the difference between G and C;
- Features for non-exact stem classification in the second stage: The same features of stage 1 plus percentage of base pairing, number of exact stems, average size of palindromes, number of internal symmetric loops, average size of internal symmetric loops, and maximum size of internal symmetric loops;
- Features for hairpin classification in the third stage: The same features of stage 2 plus average size of exact stems, size of the terminal loop, percentage of GC pairing, adjusted MFE, percentage of non-exact stems covering the hairpin, size of the biggest difference between the two sides of bulge, size of the biggest bulge to the left of the terminal loop, size of the biggest bulge to the right of the terminal loop, difference between the number of left and right bulges, number of consecutive bulges, number of consecutive bulges on the same side, MFE1, MFE 2, and triplets.

The values of MFE and deltaG are obtained from the computational tools RNAfold and RNAcofold, respectively, which are part of the Vienna RNA package [16].

### Learning with imbalanced datasets

As could be seen in the description of our data, the TSs are highly imbalanced. Namely, the number of negative instances is much greater than the number of positive instances. It may be a problem because the resulting model could be biased to the dominant class, presenting a poor accuracy to classify positive examples. In order to avoid this issue, three strategies to cope with imbalanced data were considered: Cost matrix, sampling, and the SMOTE filter [29, 30].

In cost matrices, it is possible to set the cost of misclassifying positive and negative instances. In our case, this cost is inversely proportional to the number of instances in the TS, i.e., the cost of misclassifying a positive instance, which belongs to the minority class, is much higher than the cost of misclassifying a negative instance. As a result, the weights of both classes in the training process are equalized.

Sampling and SMOTE were applied to alter the number of instances in the TS, such that the amount of instances in each class became even. To this end, sampling with replacement was used to undersampling the majority class instances, while keeping the minority class instances unchanged. The SMOTE technique, in turn, was used to oversampling the minority class elements, which eliminates the possibility of information loss. To achieve this, SMOTE combines the features of existing instances with the features of their nearest neighbors to create additional synthetic instances.

We selected a set of well known learning algorithms to be used in conjunction with the above methods for imbalanced data. Inspecting the literature about computational methods regarding learning tasks to miRNA discovery [19, 20, 31, 32], we have chosen the following learning algorithms to evaluate: SVM, multilayer perceptron (MLP), and random forest (RF). Our intention was to perform some experiments varying the combination of methods for imbalanced data with these learning algorithms to select the most appropriate approach. The results shown in the next section demonstrate that the best strategy among the possible combinations is SMOTE + RF.

To deploy all these ML methods in our computational tool, we used the API of the Weka machine learning toolkit [27], version 3.7.11, that implements all algorithms and techniques mentioned above. In particular for the SVM approach, we tested two implementations provided in the Weka API: Sequential minimal optimization (SMO) and LibSVM.

### Complexity analysis

The analyses of time and space presented here do not consider the phase of ML model construction, because it is performed once and the resulting model is applied as much as needed.

Concerning space, given an input sequence of size *n* and a sliding window of size *m*, the space required by the algorithm as a function of these variables has complexity *θ*(*n*+*m* ^2^). Notice that it is necessary to store the whole sequence and the triangular base paring matrix. For large input sequences, such as an entire chromosome or even a whole genome, it is clear that *n*≫*m*. In this case, we can consider the space complexity as *θ*(*n*).

Regarding time, for the same variables above, we can assume *n* window subsequences of size *m* to analyze, i.e., *n* executions of the three-stages pipeline, and an *m*×*m* matrix to explore in each case. Considering an extreme scenario in every stage where each of the *m* ^2^ entries of the matrix has to be processed, the matrix scanning has complexity *O*(*m* ^3^), because for each position in the matrix, it is needed *m* additional entry accesses to process the respective diagonals. Therefore, the whole algorithm time complexity is *O*(*nm* ^3^).

## Results and discussion

Experiments were performed on a Linux machine, Intel Core Duo 2 T6600, 2.2 GHz, and 3 GB of RAM. Considering we used the same platform and processor reported in the miRNAFold work, we could directly compare our running times with the times the miRNAFold authors reported for their work and for other methods.

To evaluate the ML algorithms selected for classification as well as to compare our approach with other previously proposed methods, we report sensitivity (SN) and selectivity (SL) in our experiments, which are the same statistical measures used by Tempel and Tahi [14], and are given by:

$$\begin{array}{*{20}l} SN = 100 \times \frac{TP}{(TP+FN)}, && SL = 100 \times \frac{TP}{(TP + FP)}, \end{array} $$

where TP is the number of true positives, FN is the number of false negatives, and FP is the number of false positives. Furthermore, the geometric mean (GM) of SN and SL is provided in each experiment, as suggested by Gudyś et al. for the case of imbalanced data [32].

### Deciding the ML methods to be part of Mirnacle

In order to decide the best method among the ones selected for treating the imbalance problem, all combinations of these methods with the already mentioned ML approaches were tested using TSHA1, TSHA2, and TSHA3. We kept the algorithms’ parameters with their default values [33–36].

Table 1 shows the results of this experiment in which a 10-fold cross validation was performed for each possible combination. The best GM values for each stage and for each ML algorithm are in bold in the table. Compared with other methods for imbalanced data, the SMOTE technique presented the highest GM values in all stages regardless the ML algorithm used. Even analyzing SN and SL, individually, SMOTE demonstrates better results in most cases. Still in Table 1, it can be seen that the best GM values for stages 1, 2, and 3 with SMOTE were 97.2, 98.7, and 99.8, respectively, achieved by RF, RF, and MLP, in this order. Considering that the approach SMOTE+RF produced GM = 99.7 for the third stage, which is very close to the value achieved by SMOTE+MLP, the former demonstrated to be a good choice to be implemented in Mirnacle.

**Table 1:** Comparing different combinations of methods for imbalanced data and learning algorithms. A 10-fold cross validation was performed in each case using TSHA1, TSHA2, and TSHA3 for the respective stages

Method	1st stage			2nd stage			3rd stage		
	SN	SL	GM	SN	SL	GM	SN	SL	GM
LibSVM:									
Cost matrix	4.7	64.7	17.4	3.8	81.8	17.7	4.2	100	20.6
Sampling	99.6	55.4	74.3	99.6	54.8	73.8	100	65	80.6
SMOTE	87.5	99.6	**93.4**	82.5	100	**90.8**	97.9	100	**98.9**
SMO:									
Cost matrix	80.9	17.9	38	85.6	40	58.1	97.9	77.5	87.1
Sampling	84.3	77.7	81	86	90.6	88.3	98.7	96.7	97.7
SMOTE	86.3	83.5	**84.9**	91.9	94.4	**93.1**	99.9	99.3	**99.6**
MLP:									
Cost matrix	74.1	16.7	35.2	79.2	49.7	62.7	98.7	3.9	19.6
Sampling	78.8	77.5	78.2	89.8	88.3	89.1	97	97.9	97.4
SMOTE	91.5	90.8	**91.1**	98	97.1	**97.5**	99.9	99.7	**99.8**
RF:									
Cost matrix	46.2	44	45.1	74.6	76.5	75.5	89	89	89
Sampling	84.3	78.7	81.4	87.7	87.7	87.7	97.9	97.1	97.5
SMOTE	98.2	96.3	**97.2**	99.1	98.4	**98.7**	99.9	99.4	**99.7**

Further experiments with TSHS1, TSHS2, and TSHS3 (*Homo sapiens* TSs) as well as TSMM1, TSMM2, and TSMM3 (*Mus musculus* TSs) demonstrated the same. This time, we fixed SMOTE as the technique to cope with the imbalance problem, and varied the ML algorithm. Observing the best GM values (in bold) in Table 2, it can be noticed that the SMOTE+RF approach is, in fact, a suitable choice to integrate the Mirnacle method. RF achieved the highest values for GM in all stages for both organisms, i.e., it provided the best compromise between SN and SL. MLP also showed good results in the third stage. However, it presented worse GM values for the other phases compared with RF. Moreover, the training time with MLP is much longer than with RF (not shown).

**Table 2:** Comparison of the selected classifiers combined to the SMOTE filter using TSHS1, TSHS2, and TSHS3 (A) as well as TSMM1, TSMM2, and TSMM3 (B) for the respective stages. Each ML algorithm was tested for each TS through a 10-fold cross validation

Classifier	1st stage			2nd stage			3rd stage		
	SN	SL	GM	SN	SL	GM	SN	SL	GM
(A) Results for TSHS1, TSHS2, and TSHS3:									
LibSVM	87.5	99.5	93.3	84.7	100	92	98.1	100	99.1
SMO	86.7	84	85.3	92.9	94.5	93.7	99.9	99.4	99.6
MLP	92.3	88.4	90.3	97.9	97	97.5	99.9	99.7	**99.8**
RF	97.8	97.7	**97.8**	98.7	99	**98.8**	100	99.7	**99.8**
(B) Results for TSMM1, TSMM2, and TSMM3:									
LibSVM	89.4	98.8	94	100	60.5	77.8	97.6	100	98.8
SMO	85.6	81.6	83.5	92	92.5	92.2	99.2	99	99.1
MLP	88.9	86.8	87.8	95.2	94.9	95.1	99.9	99.5	**99.7**
RF	96.4	94.6	**95.5**	97.8	97.9	**97.8**	99.8	99.6	**99.7**

### Comparing Mirnacle with other methods for pre-miRNA ab initio prediction

After defining the best ML approach to use, we could conclude our computational tool and compare it with other previously proposed methods for pre-miRNA ab initio prediction. In our experiments, only ab initio methods of the third category, mentioned earlier, were considered.

The Mirnacle parameters are: Minimum exact-stem size, sliding window size, the probability threshold of each of the three stages, minimum pre-miRNA size, and maximum pre-miRNA size. In all experiments, the minimum exact-stem size, the sliding window size, the minimum pre-miRNA size, and the maximum pre-miRNA size were set to 4, 150, 50, and 150, respectively.

Similarly to the experiments performed for miRNAFold, appropriate probability thresholds for the three stages were established using the artificially created dataset HAD. Notice that RF produces the probability of an example being positive, instead of a binary output. This is very useful because the discriminant probability can be used according to a particular goal. Hence, if sensitivity is more important, then a low threshold should be used. On the other hand, if selectivity is the priority, e.g., to minimize laboratory validations, then a high threshold is more appropriate. It is necessary to define the thresholds of each model used for each stage. For this end, we tried several combinations of thresholds (not shown) and selected five of them (represented by ordered triples) that led to different values for SN and SL: (0.3, 0.3, 0.7); (0.4, 0.4, 0.7); (0.5, 0.5, 0.7); (0.6, 0.6, 0.7); and (0.8, 0.8, 0.7). The triple (0.3, 0.3, 0.7) resulted in the best GM (Table 3) and was used to the comparisons made with datasets HSD and MMD (Table 4).

**Table 3:** Comparison of the methods for pre-miRNA ab initio prediction using sequence HAD. The results of five distinct combinations of discriminant probabilities for the three respective stages are shown in parenthesis for Mirnacle

Method	Sensitivity	Selectivity	GM	Time(mm:ss)
Mirnacle (0.3,0.3,0.7)	97	81.51	**88.91**	14:58
Mirnacle (0.4,0.4,0.7)	86	85.15	85.57	07:31
Mirnacle (0.5,0.5,0.7)	65	86.76	75.09	03:24
Mirnacle (0.6,0.6,0.7)	55	94.83	72.21	02:05
Mirnacle (0.8,0.8,0.7)	23	95.83	46.94	01:20
TVM	97	39.34	61.77	-
miRNAFold	97	19.17	43.12	00:0.84
miRPara	97	9.70	30.67	05:24
CID-miRNA	97	11.72	33.71	90:49
VMir	28	1.32	6.07	02:32

**Table 4:** Comparison of the methods for pre-miRNA ab initio prediction using sequence HSD (A) and MMD (B). The results of Mirnacle are with thresholds (0.3, 0.3, 0.7) for the three stages, respectively

Method	Sensitivity	Selectivity	GM
(A) Results for the *Homo sapiens* sequence (HSD):			
Mirnacle	100	29.52	**54.33**
TVM	100	1.43	11.95
miRNAFold	100	0.89	9.43
miRPara	98	0.93	9.54
CID-miRNA	38	0.69	5.12
VMir	100	0.56	7.48
(B) Results for the *Mus musculus* sequence (MMD):			
Mirnacle	98.61	47.33	**68.31**
TVM	100	4.13	20.32
miRNAFold	98.59	7.71	27.57
miRPara	98.59	5.34	22.94
CID-miRNA	29.58	0.82	4.92
VMir	88.73	2.93	16.12

The results shown in Tables 3 and 4 for TVM were taken from its publication [23], while the results for miRNAFold, miRPara, CID-miRNA, and VMir were obtained from the work of Tempel and Tahi [14]. Using the same criterion as the authors of miRNAFold, a predicted pre-miRNA is considered true if the distance from its center to the center of the known hairpin is less or equal to 10% of the size of the latter.

It can be seen in Table 3 that the best GM value of Mirnacle was 88.91, representing an increase of approximately 44% regarding the best result among the other methods, namely, GM = 61.77 produced by TVM. Concerning selectivity, which is the main bottleneck of the previously proposed approaches, Mirnacle could deliver a selectivity of 81.51 that means more than a two-fold increase compared with TVM. Notice that a high sensitivity value was kept by Mirnacle. In Table 3, the five results shown for different thresholds for the three stages can serve as a reference to threshold values to be used depending on a particular objective. If one is interested in high selectivity to reduce biological validations, the combination (0.8,0.8,0.7) is a good option, for instance.

Concerning the running time, Mirnacle’s performance was highly variable. This is because low thresholds in the first and the second stages mean a less strict filter of exact and non-exact stems, i.e., the third stage will contain more sequences to extend to a complete hairpin, which leads to a longer running time. High thresholds in the first and second stages, on the other hand, mean fewer non-exact stems to expand at the end, speeding up the process. Notice that the matrix exploration in the third phase for inspecting different possibilities of a complete hairpin is the most computationally-expensive part. Comparing the Mirnacle execution that took 14 minutes and 58 seconds with the running time of other methods, Mirnacle could only overcome CID-miRNA. However, considering that we substantially improved selectivity, the time saved in laboratory experiments is likely more significant. It can be seen that the time taken by TVM was not reported. The reason for this is that the authors do not mention any experiment to measure time. Furthermore, TVM is available only as a webserver, which makes infeasible to measure its running time in a fair way.

Table 4 shows that Mirnacle provided even more significant improvements in selectivity for the other datasets. It can be seen an increase of 20-fold compared with TVM for sequence HSD (Table 4A), and an increase of 6-fold compared with miRNAFold for sequence MMD (Table 4B), while maintaining high sensitivity. The results for sequence HSD are particularly remarkable. For sequence MMD, Mirnacle missed one pre-miRNA, resulting in slightly lower sensitivity compared with TVM. However, the improvement in selectivity produced by Mirnacle led to a much higher GM value of this method.

## Conclusions

In this work, we propose an extension of the miRNAFold method for pre-miRNA ab initio prediction to address the low selectivity issue of miRNAFold and other ab initio approaches. Our experiments have shown that our method, termed Mirnacle, substantially increased the selectivity compared with previously proposed procedures, whereas keeping high sensitivity.

To achieve these results, the main improvements implemented in Mirnacle were: The analysis of negative training examples, in addition to positive examples; the use of machine learning techniques, namely, SMOTE combined with random forest; and the inclusion of other important hairpin features. Furthermore, Mirnacle allows the user to provide positive and negative examples to generate new models, which results in great flexibility in the use of our computational tool, i.e., as long as appropriate training examples are available, Mirnacle can be, in principle, used for other organisms of interest.

As a future work, we intend to improve Mirnacle’s running time. First, the calculation of MFE and deltaG will be part of Mirnacle’s code, in order to eliminate calls to the Vienna RNA package. Second, and most importantly, Mirnacle will implement parallel approaches, such as GPU (graphics processor unit), as the analyses of the subsequences in a genome are completely independent. Therefore, the parallelization of such analyses can certainly bring a huge gain in performance.

## Abbreviations

API: Application programming interface
FP: False positive
GM: Geometric mean
GPU: Graphics processor unit
HAD: Human artificial data
HSD: *Homo sapiens* data
miRNA: MicroRNA
ML: Machine learning
MMD: *Mus musculus* data
MFE: Minimum free energy
MLP: Multilayer perceptron
miscRNA: Miscellaneous RNA
NGS: Next-generation sequencing
pre-miRNA: miRNA precursor
pri-miRNA: primary miRNA
RF: Random forest
SVM: Support vector machines
snRNA: Small nuclear RNA
snoRNA: Small nucleolar RNA
SMO: Sequential minimal optimization
SN: Sensitivity
SL: Selectivity
TS: Training set
