# Erratum to: Mirnacle: machine learning with SMOTE and random forest for improving selectivity in pre-miRNA ab initio prediction

**DOI:** 10.1186/s12859-017-1508-0

**Published:** 2017-02-17

**Authors:** Yuri Bento Marques, Alcione de Paiva Oliveira, Ana Tereza Ribeiro Vasconcelos, Fabio Ribeiro Cerqueira

**Affiliations:** 1 Department of Informatics, Universidade Federal de Viçosa, Viçosa, 36570-900 Brazil; 2 Instituto Federal do Norte de Minas, Rua Mocambi, Teófilo Otoni, 39800-430 Brazil; 3 Department of Computer Science, University of Sheffield, Western Bank, S10 2TN Sheffield, UK; 4 Laboratório Nacional de Computação Científica, Rua Getúlio Vargas 333, Petropólis, 25651-071 Brazil

## Erratum

The authors of this published article [1] would like to correct their ‘Funding’ declarations from: “Publication charges for this article have been funded by CAPES, CNPq, and FAPEMIG” to: "The publication charges for this article were funded by CAPES (process no. 23038.010041/2013-13 - Aux. 3384/2013 - Biocomputacional)".
